# Supplementary material for: Hybrid Models and Biological Model Reduction with PyDSTool
Source: PLoS Comput Biol. 2012 Aug 9;8(8):e1002628. doi: 10.1371/journal.pcbi.1002628 (PMC3415397; doi:10.1371/journal.pcbi.1002628)
Supplement: Text S4 — Complete source code for the PyDSTool package (version 0.88.120504). Includes API documentation and help files linking to web pages. This file is identical to the current public release on Sourceforge.net. (ZIP) [file pcbi.1002628.s004.zip › PyDSTool/html/PyDSTool.Toolbox.optimizers.helpers.finite_difference.ForwardFiniteDifferencesCache-class.html]

xml version="1.0" encoding="ascii"?


PyDSTool.Toolbox.optimizers.helpers.finite\_difference.ForwardFiniteDifferencesCache


| Home | Trees | Indices | Help | PyDSTool |
| --- | --- | --- | --- | --- |

Package PyDSTool ::
Package Toolbox ::
Package optimizers ::
Package helpers ::
Module finite\_difference ::
Class ForwardFiniteDifferencesCache

[hide private]

[frames] | no frames]

# Class ForwardFiniteDifferencesCache

source code

```
               object --+        
                        |        
FiniteDifferencesFunction --+    
                            |    
       FiniteDifferencesCache --+
                                |
                               ForwardFiniteDifferencesCache
```

Known Subclasses:
:   - ParamEst.residual\_fn\_context
    - , ParamEst.residual\_fn\_context\_1D

---

A function that will be able to computes its derivatives with a
forward difference formula.


|  |  |
| --- | --- |
| Instance Methods | [hide private] |

|  |  |
| --- | --- |
| jacobian(self, params, extra\_args=None)  Computes the jacobian of the function | source code |

|  |  |
| --- | --- |
| gradient(self, params)  Computes the gradient of the function | source code |

|  |  |
| --- | --- |
| hessian(self, params)  Computes the hessian of the function approximated by J^T \* T | source code |

**Inherited from `FiniteDifferencesCache`**:
`__call__`,
`__init__`,
`residual`

**Inherited from `FiniteDifferencesCache`** (private):
`_res_fn`

**Inherited from `object`**:
`__delattr__`,
`__getattribute__`,
`__hash__`,
`__new__`,
`__reduce__`,
`__reduce_ex__`,
`__repr__`,
`__setattr__`,
`__str__`


|  |  |
| --- | --- |
| Properties | [hide private] |

**Inherited from `object`**:
`__class__`

| Home | Trees | Indices | Help | PyDSTool |
| --- | --- | --- | --- | --- |

|  |  |
| --- | --- |
| Generated by Epydoc 3.0.1 on Fri May 4 15:24:09 2012 | http://epydoc.sourceforge.net |
